# Effect of antiplatelet agents on *Escherichia coli* sepsis mechanisms: A review

**DOI:** 10.3389/fmicb.2022.1043334

**Published:** 2022-12-09

**Authors:** Antoine Mariotti, Amina Ezzeroug Ezzraimi, Laurence Camoin-Jau

**Affiliations:** ^1^Aix Marseille Univ., IRD, APHM, MEPHI, IHU Méditerranée Infection, Marseille, France; ^2^IHU Méditerranée Infection, Marseille, France; ^3^Haematology Laboratory, Hôpital de la Timone, APHM, Marseille, France

**Keywords:** *Escherichia coli*, platelets, platelet aggregation, TLR4, antiplatelet drugs, sepsis

## Abstract

Despite ever-increasing improvements in the prognosis of sepsis, this condition remains a frequent cause of hospitalization and mortality in Western countries. Sepsis exposes the patient to multiple complications, including thrombotic complications, due to the ability of circulating bacteria to activate platelets. One of the bacteria most frequently implicated in sepsis, *Escherichia coli*, a Gram-negative bacillus, has been described as being capable of inducing platelet activation during sepsis. However, to date, the mechanisms involved in this activation have not been clearly established, due to their multiple characteristics. Many signaling pathways are thought to be involved. At the same time, reports on the use of antiplatelet agents in sepsis to reduce platelet activation have been published, with variable results. To date, their use in sepsis remains controversial. The aim of this review is to summarize the currently available knowledge on the mechanisms of platelet activation secondary to *Escherichia coli* sepsis, as well as to provide an update on the effects of antiplatelet agents in these pathological circumstances.

## Introduction

In addition to their role in hemostasis, platelets play a major role in the anti-infective response and in the regulation of the inflammatory response (Thomas and Storey, 2015). This anti-infective defense role of platelets has been demonstrated by their ability to interact and activate in the presence of many classes of pathogens. They are involved in antiviral defense, notably through the release of the chemokine CCL5, promoting the development of a protective response during dengue virus (Singh et al., 2020; Tokarz-Deptuła et al., 2021) and hepatitis C virus (HCV) infection (Katsounas et al., 2011). More recently, platelets have also been described to be involved in a deleterious response during SARS-CoV-2 infection, linked to abnormal expression of certain genes (Manne et al., 2020), making platelets hyper-reactive and promoting the procoagulant state found in critical patients with COVID-19 (Campbell et al., 2021; Zhu et al., 2022).

Platelet activation may also play an important role in the pathophysiological mechanisms of certain parasitic infections, such as malaria, where platelets are a key player in the neurological complications of malaria due to their ability to form microthrombi. This response initially limits parasite proliferation and has a protective effect on the host, but will later become deleterious if platelet activation persists (Aggrey et al., 2013).

Many receptors located on the surface of platelets have been shown to be involved in the interaction with bacteria, such as TLRs, the PAF receptor, FcγRIIA or GPIbα (Cox et al., 2011). During sepsis, bacteria will be able to interact with one or more of these receptors and induce platelet activation that can lead to the appearance of deleterious phenomena, such as the appearance of thrombosis or deregulated inflammation, or beneficial, with a demonstrated bactericidal effect of platelets on certain bacterial strains (Ezzeroug Ezzraimi et al., 2022b).

Sepsis is characterized by complex pathological mechanisms and is associated with a high mortality rate (Gotts and Matthay, 2016). In 1991, a consensus conference proposed the initial definition of sepsis as “a syndrome of systemic inflammatory response (SIRS) of the host to an infection” (Bone et al., 1992). In 2016, a new definition of sepsis was developed as “life-threatening organ dysfunction caused by a dysregulated host response to infection” (Singer et al., 2016). Sepsis is an extremely serious condition in which bacteria induce the activation of hemostasis and, in particular, the activation of platelets across the entire vascular system, leading to the phenomena of immuno-thrombosis (Martinod and Deppermann, 2021), which is based on an uncontrolled interaction of the systems of inflammation and hemostasis, with platelets being an integral part of both systems.

In this review, we will focus on *Escherichia coli* sepsis. We will review the current state of knowledge on the mechanisms of interaction between platelets and *Escherichia coli*, and the potential value of antiplatelets in this indication.

## Platelets in the pathophysiology of sepsis

### Platelet–bacteria interactions during sepsis

In recent years, a growing number of studies have demonstrated that platelets are involved in the deleterious processes observed during sepsis and that they play an important role in the development of organ damage that can lead to multiple organ dysfunction syndrome (MODS; Greco et al., 2017).

In the event of vascular invasion, bacteria enter the bloodstream, which triggers defense mechanisms. Activation of hemostasis at the site of the injury and the formation of thrombi in local capillaries not only stops bleeding, but also initiates an early anti-infective response. Platelets will express receptors (P-selectin, CD40L or CD154) allowing interaction with immune cells or with endothelial cells (*via* PSGL-1, CD154 receptors) which allow signal transduction and activation of these different cell types (Koupenova et al., 2018). This phenomenon is a defense mechanism that limits infection of the lesions by a process known as immunothrombosis or thromboinflammation (Martinod and Deppermann, 2021). In the case of sepsis, local reactions extend to the whole body, producing deleterious phenomena in many tissues. Thus, in an animal model of abdominal sepsis, neutrophil infiltration of the lung, induced by platelet activation, is thought to contribute towards the development of pulmonary edema (Asaduzzaman et al., 2009).

**Figure 1:**
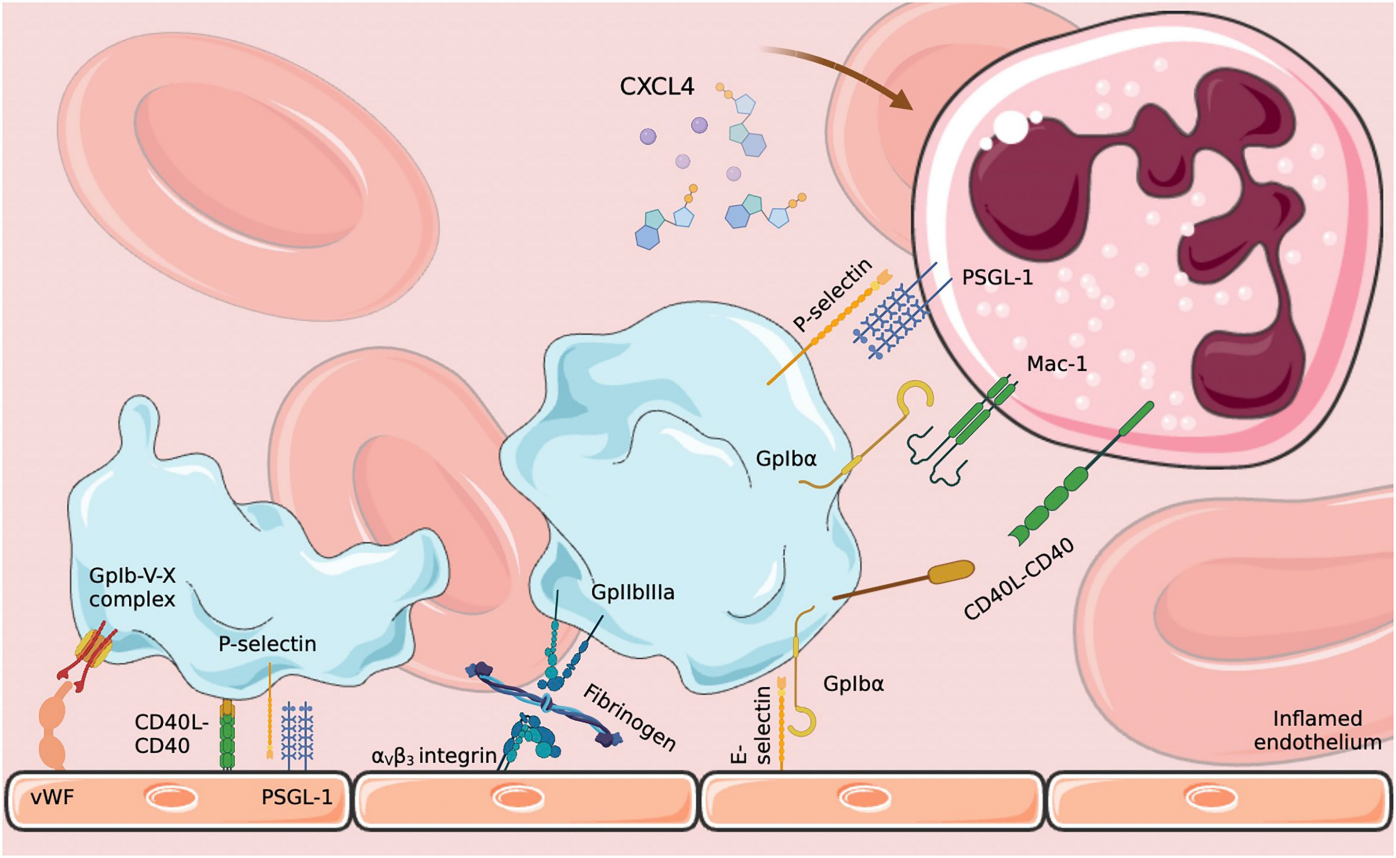
Platelet–endothelium and platelet–neutrophil interactions. Endothelial activation in response to infection induces platelet activation which in turn activates neutrophils through various signaling pathways. CXCL4: platelet factor 4; PSGL-1: P-selectin glycoprotein 1; E-selectin: endothelial selectin. Diagram created on biorender.com using SMART (Servier Medical Art).

At the same time, endothelial activation, observed during sepsis, leads to the appearance of or increase in surface molecules, such as von Willebrand factor (vWF), E-selectin and integrins α_V_β_3_, encouraging interaction with platelets and leading to their activation, while decreasing anti-adhesive inhibition pathways, thus favoring the risk of thrombosis (Romo et al., 1999; Kaplan and Jackson, 2011; Figure 1). Ischemia in several areas, secondary to the appearance of generalized activation of endothelial cells, may be observed, leading in particular to abnormalities in blood pressure and vascular permeability (Opal and van der Poll, 2015) associated with the formation of micro clots.

Several types of signaling mechanisms and pathways may be involved depending on the bacterial species, or on the receptor implicated in this interaction.

### Platelets and immune cells

Platelets can therefore exhibit several effects that will take place during sepsis in a chronological manner. Firstly, they will be involved in the recognition of specific bacterial patterns, notably through TLRs (Shiraki et al., 2004; Andonegui et al., 2005). Once the bacteria have been recognized, a platelet response will occur, which will vary according to several parameters: the bacterial species involved and their escape mechanisms, and the platelet receptors and signaling pathways involved. Platelets can therefore be involved in the destruction of pathogens, either by direct cytotoxicity (Kraemer et al., 2011; Ezzeroug Ezzraimi et al., 2022b) or by cooperation with other cell types, through opsonisation (Semple et al., 2007) or NETosis (Clark et al., 2007; McDonald et al., 2012). By activating, platelets will also cause the release of chemical mediators (CCL5, CXCL4), which will be involved in chemotaxis of certain immune cells, notably neutrophils and monocytes (Goncalves et al., 2011; Sreeramkumar et al., 2014), but also in the modulation of the inflammatory response (Bakogiannis et al., 2019). This interaction with the inflammatory system will take place *via* certain cytokines (Brown et al., 2013) or *via* the complement system (Nording and Langer, 2018; Palm et al., 2019). Platelets will also be able to interact with the coagulation system and remain a major player in the initiation of DIC and the procoagulant state encountered during sepsis (Delabranche et al., 2017; Yang et al., 2017). In fact, recent studies show that there are platelet subpopulations, including pro-coagulant platelets that arise in response to intraplatelet calcium release. These pro-coagulant platelets will play a key role in the regulation of thrombotic and hemorrhagic phenomena, but also in thromboinflammation and NETosis, by interacting with certain immune cells, notably neutrophils (Denorme and Campbell, 2022).

The deregulation of the NETosis phenomenon, encountered during sepsis, may lead to the development of deleterious effects, relying on unregulated activation of neutrophils in response to a platelet-derived signal to scavenge circulating bacteria (Clark et al., 2007). However, the formation of these NETs will also lead to the formation of a pro-coagulant terrain allowing the attachment of certain coagulation factors or extracellular vesicles (Zaid and Merhi, 2022), which can favor the development of arterial and venous thrombotic phenomena (Martinod and Wagner, 2014). These deleterious effects, when prolonged, can lead to either localized organ dysfunction or to multiple organ failure syndromes. The organs most frequently concerned are the kidneys, through the development of acute renal failure secondary to renal hypoperfusion, but also secondary to endothelial damage (Gómez and Kellum, 2016); the liver, which is the site of the synthesis of numerous cytokines, and which can therefore play an important role in the anarchic inflammation that occurs during sepsis (Yan et al., 2014); and the circulatory system, notably through the systemic activation of endothelial cells and the significant release of vasodilator molecules such as nitric oxide (NO), leading to hypotension that is almost always encountered during sepsis (Vincent et al., 2000).

Some bacteria also appear to have escape mechanisms from the platelet-induced anti-infective response. *Yersinia pestis*, for example, is able to induce a change in the structure of the thrombus, formed by platelets and fibrin, in order to escape the NETosis phenomenon. This is possible through one of its virulence factors (Y pestis plasminogen activator Pla) which activates a fibrinolysis phenomenon, allowing an escape from bactericidal action (Palace et al., 2020).

### Other actors of thrombosis in sepsis

However, platelets are not the only factors linking hemostasis and inflammation. Certain mediators of inflammation have the ability to interact with different factors in the coagulation cascade, creating a pro-thrombotic state. The coagulation and complement systems, usually represented separately, are in fact closely intertwined. Proteins involved in one of the cascades are capable of interacting with factors in the other system. For example, the activation of factor XII to activated factor XIIa is capable of inducing the activation of the classical complement pathway, while C4-binding protein (C4BP) can bind to protein S and inhibit its effect, thereby promoting the development of thrombosis (Rittirsch et al., 2008). The existence of exacerbated inflammation can therefore lead to the development of hypercoagulability and, in the most severe cases, induce the onset of disseminated intravascular coagulation (DIC), thus increasing the risk of developing tissue hypoxia and organ dysfunction through the formation of thrombi in the capillary circulation (Levi and Ten Cate, 1999; Donzé et al., 2014).

In summary, this inflammation-coagulation phenomenon in sepsis, associated with endothelial damage, is partly the result of the activation of platelets, which are able, through some of their membrane receptors, to participate in anti-infective defense. Platelets, once activated, will exacerbate systemic inflammatory reactions and coagulation disorders through interactions with immune cells and endothelial cells. The platelet activation observed during sepsis could also partly explain the thrombocytopenia frequently observed during sepsis, through a consumption mechanism (Larkin et al., 2016). In addition to the already high mortality rate in sepsis, linked to the intrinsic severity of the disease, the occurrence of thrombocytopenia further worsens the prognosis, exposing the patient to higher morbimortality (Vanderschueren et al., 2000). This includes an increased risk of bleeding, the development of acute renal failure, a longer stay in intensive care, and even mortality if the thrombocytopenia is not resolved (Venkata et al., 2013).

Thus, the inhibition of platelet activation may reduce uncontrolled inflammatory and coagulation reactions in sepsis, thereby reducing the severity of organ damage and improving patient prognosis (Dewitte et al., 2017).

## *Escherichia coli* sepsis

A 2021 meta-analysis studying the epidemiology of bacteremia-causing agents between 2007 and 2018 in Western countries estimates that *Escherichia coli* is found in an average of 27.1% of bacteremia cases, although there is considerable heterogeneity between studies, ranging from 6.5 to 57% (Bonten et al., 2021). The main entry point identified was the urogenital tract, responsible for more than 50% of infections. According to this meta-analysis, the overall incidence of *Escherichia coli* bacteremia, all groups combined, is 40.2–57.2 per 100,000 inhabitants per year, with a mortality rate of between 2.9 and 10.3 per 100,000 people. On a smaller scale, a 2019 United Kingdom report indicated that the incidence rate of *Escherichia coli* bacteremia has been increasing over the past 10 years, with a significant acceleration since 2014, from 55.2 per 100,000 population in 2014–70.7 cases per 100,000 population (Heal Protection Report, 2019).

However, these figures remain global statistics, and should be put into perspective according to the sex and age of the patients as well as their underlying pathologies. The incidence is higher in women than in men, and increases sharply with age, with statistically higher rates in the general population from the age of 60. Indeed, the incidence is multiplied by 30 in subjects over 75 years of age compared to young adults (Semple et al., 2007). The subgroup analysis in this meta-analysis shows that patients with hematological malignancies are most at risk of developing *Escherichia coli* sepsis. A study in a Swedish center found a prevalence of up to 12.7% in patients with chronic lymphocytic leukemia (Kjellander et al., 2016). Among other hematological diseases, *Escherichia coli* is responsible for 46% of bacteremia’s in acute leukemia (Cattaneo et al., 2014) and 22.4% in multiple myeloma (Teh et al., 2017). In addition, *Escherichia coli* is found in 34.2% of neutropenic patients with sepsis (Trecarichi et al., 2015). *Escherichia coli* is also found in patients with solid cancers. In a 2014 study, it was implicated in 30.5% of bacteremia’s (Marín et al., 2014) and more precisely in 22.2% of patients with colon cancer (Belhassen-García et al., 2013). Finally, surgical patients are also at risk of developing *Escherichia coli* sepsis, particularly those who have undergone abdominal surgery, due to the important localization of this pathogen in the digestive tract. *Escherichia coli* was implicated in more than a quarter of cases of sepsis after pancreatic resection and in 12.4% of gastric resections (Jannasch et al., 2015). These figures are consistent with those of another retrospective study on the development of septic shock after digestive surgery, where *Escherichia coli* was found in 16.8% of cases (Hizette et al., 2009).

However, cancer is only the third most common relative risk for developing *Escherichia coli* bacteremia (RR: 14.9). Indeed, this relative risk is 26.9 for patients with renal failure on dialysis and 20.for patients with solid organ transplantation (Bonten et al., 2021).

*Escherichia coli* sepsis also affects other categories of patients. A retrospective study carried out in Ireland showed that between 2001 and 2014, *Escherichia coli* was involved in 37% of cases of sepsis in pregnant women, a population particularly at risk, as sepsis accounts for a quarter of maternal deaths in pregnancy (Drew et al., 2015). Similarly, neonates are a particularly high-risk patient group. Although Streptococcus B (*Streptococcus agalactiae*) is the most frequently implicated germ in newborn sepsis (38–43% of cases), *Escherichia coli* sepsis is the second most important cause of mortality (24% of all episodes), being implicated in 24.5% of sepsis-related deaths (Weston et al., 2011). This high mortality is partly explained by the fact that 81% of *Escherichia coli* bacteremia occur in premature infants, who are at greater risk of infection due to their as yet fragile immunity (Simonsen et al., 2014).

**Figure 2:**
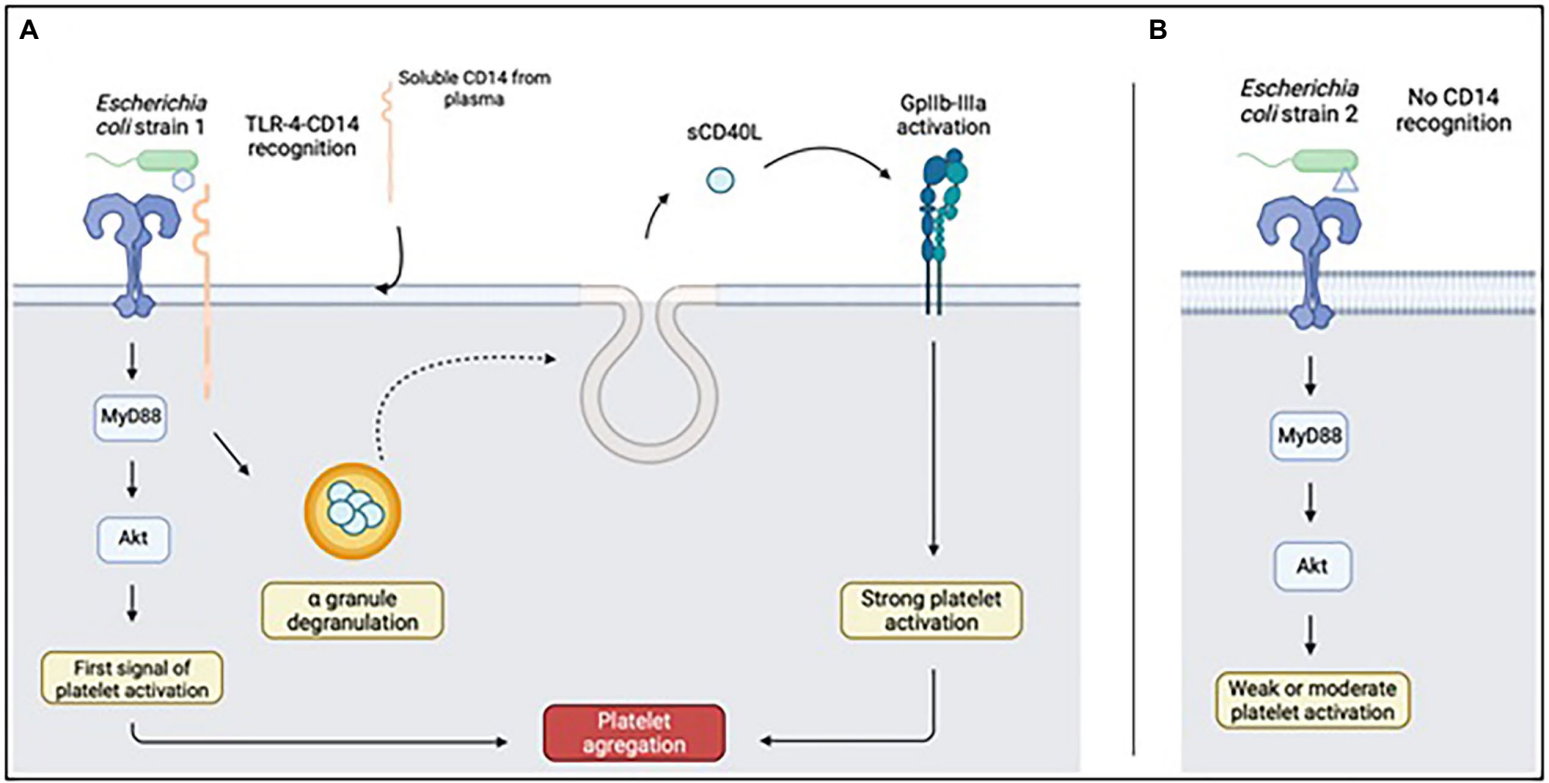
Platelet response to LPS recognized **(A)** or not recognized **(B)** by CD14. Upon full recognition of the LPS from the Escherichia coli strain, TLR4 will be able to complex with CD14 of soluble origin and be captured by platelets. This mechanism will result in the release of sCD40L present in platelet alpha granules, capable of activating GpIIbIIIa and responsible for platelet aggregation. Diagram created on biorender.com; sCD40L: soluble CD40L.

### *Escherichia coli*: General information, classification and pathogenicity

*Escherichia coli* is a Gram-negative commensal bacterium belonging to the Enterobacteriaceae family, frequently found in the human digestive tract and representing a large part of the intestinal flora. Certain strains are often found in human pathologies, particularly in community and nosocomial infections, in a wide variety of sites, including meningitis, gastroenteritis, and urinary tract infections (Kaper et al., 2004).

These *Escherichia coli* strains are capable of acquiring virulence factors (adhesins, capsule, synthesis and secretion of toxins, etc.) which confer their pathogenic power, as well as antibiotic resistance mechanisms which give them reduced sensitivity to certain anti-infective molecules (Lüthje and Brauner, 2014; Poirel et al., 2018).

**Figure 3:**
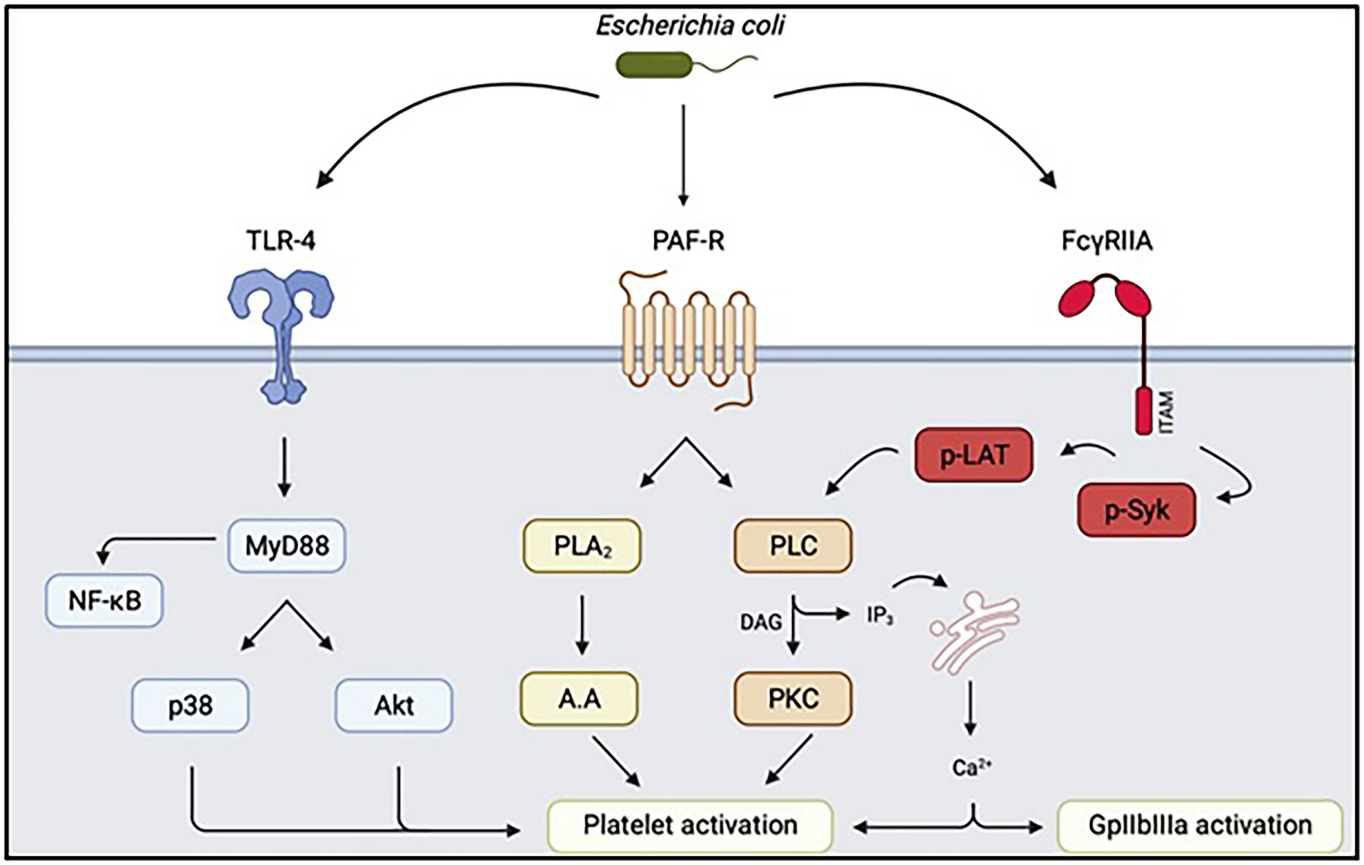
Summary of intra-platelet signaling pathways secondary to interaction with Escherichia coli. Depending on the strain involved, the signaling pathways involved in triggering platelet activation and aggregation may be different. TLR4: Toll-like Receptor 4, PAF-R: Receptor of Platelet Activating Factor, FcγRIIA: Platelet Fcγ receptor, Akt: protein kinase B, PLA2: phospholipase A2, PLC: phospholipase C, p-LAT: phospho-LAT, PKC protein kinase C. Diagram created on biorender.com.

In 2016, a classification of *Escherichia coli* strains into several subclasses was proposed (Vila et al., 2016) according to the syndromes they are capable of causing, which themselves depend on the different virulence factors that the strain may have acquired. A distinction must be made between enterohaemorrhagic (EHEC), enterotoxigenic (ETEC), enteropathogenic (EPEC) and enteroinvasive (EIEC) strains, which are similar to Shigella. These pathogenic strains all have a tropism for the digestive tract and will cause gastrointestinal manifestations. Commensal strains, on the other hand, are only rarely pathogenic, in cases of extra-intestinal dissemination linked to particular circumstances (major immunodepression, abdominal trauma, etc.). Finally, it is important to distinguish strains that cause extra-intestinal pathogens (ExPEC), which can reach many organs, but which are often found as commensals of the digestive tract. These strains have a particular ability to disseminate and survive in a normally sterile site, leading to colonization and potential infection in these extra-intestinal locations.

However, although *Escherichia coli* is frequently encountered in clinical practice, very few studies have investigated the mechanisms of *Escherichia coli*-platelet interactions. Despite a growing interest in Gram-negative bacilli, including *Escherichia coli*, studies on bacterial interactions leading to platelet activation have been mainly limited to Gram-positive bacteria, including staphylococci [*Staphylococcus aureus* and *Staphylococcus epidermidis* (Herrmann et al., 1993; Bayer et al., 1995)] and Streptococci [*Streptococcus sanguinis* and *Streptococcus gordonii* (Kurpiewski et al., 1983; Keane et al., 2010)].

## Platelet–*Escherichia coli* interactions

Three mechanisms could explain the interaction between bacteria and platelets (Cox, 2009; Cox et al., 2011) responsible for platelet activation. Bacteria can bind to platelets *via* a plasma protein. This is the case for *Staphylococcus aureus* and *Helicobacter pylori*, which are able to bind to vWF. Bacteria can direct binding to a platelet receptor. *Streptococcus gordonii* and *Streptococcus sanguinis* can directly interact with GpIb *via* their Hs antigen. This interaction can also be mediated by secreted bacterial proteins, i.e., toxins.

When activated, platelets secrete the contents of their granules, which contain more than 300 molecules (Sharda and Flaumenhaft, 2018) including adenosine diphosphate (ADP) and serotonin. Secreted cytokines and chemokines recruit leukocytes, and secreted antimicrobial peptides act to kill pathogens. This ability to activate in response to infection thus gives them the ability to destroy bacteria through bactericidal activity (Ezzeroug Ezzraimi et al., 2022b). This demonstrates that their activation and degranulation play an important role in the fight against infection.

The presence of multiple mechanisms makes it difficult to identify the roles of different proteins (both bacterial and platelet; Table 1). This analysis is further complicated by the fact that interactions are not only species-specific but also strain-specific, as demonstrated in 2016 by Watson et al. (2016) and Ezzeroug Ezzraimi et al. (2022a,b). Some interactions lead to platelet activation, while others will have no direct effect. One study even highlights the fact that LPS promotes a dose-dependent decrease in platelet reactivity in response to certain agonists, notably thrombin or ADP. This inhibition of platelet aggregation would be due to modifications in the concentration of numerous substances, such as thromboxane A_2_ or cyclic GMP (Sheu et al., 1998).

Non-activating interactions are generally of high affinity and probably play a role in supporting platelet adhesion under the shear conditions encountered in the circulation (Roka-Moiia et al., 2020). Typically, the bacterial proteins involved in adhesion are distinct from those that induce aggregation. Thus, bacteria can promote platelet adhesion and/or trigger platelet activation. Platelet activation is characterized by the appearance of or increase in certain platelet surface markers, or by the secretion of granular content [CD42b, P-selectin (CD62P) and activated GpIIbIIIa (CD41)]. These markers are most often detected by flow cytometry, but do not necessarily indicate the formation of a platelet aggregate (Shannon, 2017).

One model in which the relationship between *Escherichia coli* and platelets has been well described is hemolytic uremic syndrome (HUS), which is characterized by, among other things, mechanically induced hemolytic anemia and thrombocytopenia due to activation of platelets by altering the vascular endothelium caused by the production of Shigatoxins (Petruzziello-Pellegrini et al., 2013). Work on the *Escherichia coli* O111 strain has led to a better understanding of these interactions. This strain of *Escherichia coli* O111 producing these Shigatoxins could interact directly with platelets *via* Toll-like Receptor 4 (TLR4), leading to an increase in platelet activation markers and the expression of tissue factor (Matus et al., 2017). The complement system, as well as variations in the LPS O antigen, could explain the observed platelet activation (Zhao et al., 2002). However, these studies did not establish the predominant interaction mechanism for all *Escherichia coli* strains, which appears to be strain-dependent. Indeed, the *Escherichia coli* O157:H7 strain, which is also responsible for HUS, is thought to induce FcγRIIA receptor-mediated platelet aggregation rather than TLR4 (Moriarty et al., 2016).

Although as early as 1971, a paper demonstrated the ability of *Escherichia coli* to induce platelet aggregation (Clawson and White, 1971), it was not until several decades later that the mechanisms of this platelet aggregation were studied and that some interest was shown in other strains of *Escherichia coli*. The study by Watson et al. identified two important characteristics. The platelet aggregation they are able to induce is dependent on both the strain involved, as well as the platelet/bacteria ratio, demonstrating that the concentration of the inoculum also plays an important role. This platelet aggregation responds to the “all or nothing” law, which means that a given strain will either be able to induce significant platelet aggregation or will have no effect. In this study, the key role of FcγRIIA and integrin αIIbβ3, also known as GpIIbIIIa (Watson et al., 2016) is also described, although, as we have seen, these signaling pathways may play a different role depending on the strains involved. This notion was confirmed by Fejes et al. on the reference strain K12, which is reported to induce an elevation of the platelet activation markers P-selectin, CD63, the GPIIbIIIa activation marker PAC-1, and bound fibrinogen (Fejes et al., 2018). In the paper by Fejes et al., *Escherichia coli* strains were classified according to the structure of their lipopolysaccharide (LPS), specifically lipid A, which is thought to partly determine their interaction with platelets as well as with certain immune system cells. Some Gram-negative bacteria have a ‘smooth’ LPS, such as *Escherichia coli* O18:K1, while others have a ‘rough’ LPS, such as *Escherichia coli* K12 strains (Pupo et al., 2013). Strains with a ‘rough’ LPS would activate a wider spectrum of cells including macrophages, and with greater efficiency, than strains with a ‘smooth’ LPS (Fejes et al., 2018). Thus, this ability or inability to aggregate platelets could depend on the structure of the LPS of the Gram-negative bacteria, and their ability or inability to be recognized by the TLR4 expressed on the surface of platelets. The O antigen may also play an important role in the recognition of these bacterial patterns by platelets and in the induction or non-induction of septic shock (Zhao et al., 2002). The serotype of the strain involved in an infection in human pathology could thus play an important role in the prognosis of the patient.

The demonstration of TLR expression, particularly TLR4, on the surface of platelets has confirmed the hypothesis of a role for platelets in the anti-infective response (Vallance et al., 2017). The interaction between LPS from Gram-negative bacteria and platelets will lead to an increase in platelet binding to fibrinogen in a possibly TLR4-dependent manner (Fejes et al., 2018). Another major consequence of the presence of these TLRs on the platelet surface is the ability of platelets to be sequestered in the lung through their interaction with neutrophils (Andonegui et al., 2005).

The signaling pathways secondary to the interaction between LPS and TLR4 have been extensively studied to determine whether these receptors were indeed responsible for the platelet activation observed upon contact with *Escherichia coli*. The various platelet TLRs were able to induce, *via* activation of MyD88 (Zhang et al., 2009; Berthet et al., 2010) leading to activation of the PI3K/Akt pathway, leading to platelet activation and subsequent platelet aggregation. LPS, by interacting with TLR4, would not by itself lead to platelet activation, but would play a facilitating role in platelet adhesion, in association with a platelet agonist, and would also promote platelet secretion and aggregation (Zhang et al., 2009; Niklaus et al., 2020). It thus appears that *Escherichia coli* LPS, depending on the strain used, has a variable capacity to induce platelet aggregation. Similarly, in addition to its effect on platelet TLR4, the ability of *Escherichia coli* LPS to interact with TLR4 of other cell types could induce the production of extracellular microvesicles with a strong procoagulant potential, which may partly explain the risk of DIC in infected patients (Wang et al., 2019). In addition to being involved in the synthesis of microvesicles, platelets are capable of internalizing their own microvesicles. This ability of platelets to endocytose microvesicles is thought to be dependent on the TLR4 receptor, and to promote the development of a prothrombotic state (Jerez-Dolz et al., 2020). However, platelets are not the only actors involved in the release of procoagulant microvesicles. The role of monocyte-derived microvesicles, containing significant amounts of tissue factor, in the development of thrombotic complications of sepsis has also been demonstrated (Morrissey and Drake, 1993; Grover and Mackman, 2018).

TLR4 would not recognize LPS from different strains in the same way. Some forms would be able to bind only the TLR4 receptor, while others would induce the formation of a TLR4-sCD14 complex, which would involve specific signaling pathways (Berthet et al., 2012). Since platelets do not produce sCD14, it would be adsorbed onto the platelet surface from the plasma (Damien et al., 2015). This effect would be very specific to activation by LPS and would not be observed with conventional agonists such as TRAP. CD14 plays a critical role in the physiopathology of sepsis. Its inhibition would attenuate the deleterious responses linked to pro-inflammatory cytokines and reduce the procoagulant state that accompanies sepsis (Thorgersen et al., 2010; Keshari et al., 2021).

Knowledge of this second activation pathway by the TLR4-sCD14 complex is particularly important, since it would lead to the release of CD40L contained in platelet granules (Damien et al., 2015) which would be able to induce or promote platelet aggregation, by having some affinity for integrin αIIbβ3 (Davì and Ferroni, 2005) integrin or by raising thromboxane A_2_ levels (Kojok et al., 2020; Figure 2). LPS is thought to cross-react with the PAF receptor, PAF-R (Figure 3). PAF is an agent capable of causing intense platelet activation and disseminated intravascular coagulation syndromes very rapidly after injection in mice, leading to the death of the animal (Abhilasha et al., 2019). The ability of LPS to induce activation of the PAF-R receptor has been known for many years. Indeed, LPS increases the expression of PAF-R *in vitro*, while potentiating its effect, even if the effects *in vivo* were more moderate (Wang et al., 1997). Furthermore, LPS, in addition to PAF-R-mediated platelet aggregation, is capable of inducing a tolerance phenomenon and thus of decreasing the response of platelets to PAF in the event of prior exposure, as well as modifying the expression of certain genes coding for pro-inflammatory cytokines (Abhilasha et al., 2021). These different properties of LPS thus show that bacteria, through their structural proteins, are able to induce a complex platelet response mediated by numerous signaling pathways. This effect described on PAF is particularly interesting. Indeed, although the percentage of platelet aggregation measured by aggregometry decreased in response to antiplatelet drugs, there was still an ability of platelets to aggregate in the presence of PAF. This residual aggregation is not observed when platelets are activated by ADP (Riaz et al., 2012).

FcγRIIA may also play a role in platelet activation (Figure 3). The anti-infective role of platelets is thought to be partly dependent on this receptor, their bactericidal activity being linked to the recognition of IgG deposited on bacteria during opsonisation (Riaz et al., 2012). Therefore, these IgGs, *via* the Fc fragment, will be recognized by FcγRIIA and induce a platelet response capable of killing these bacteria. This FcγRIIA-dependent pathway is also involved in the platelet aggregation mechanism when platelets are exposed to bacteria, through the formation of immune complexes (Arman and Krauel, 2015). Contact of a strain of *Escherichia coli* responsible for HUS (O157:H7) with platelets would trigger strong platelet aggregation, which would be completely inhibited in the presence of an anti-FcγRIIA (Moriarty et al., 2016). In contrast, the aggregation induced by this strain of *Escherichia coli* is not dependent on the TLR4 signaling pathway. The multiplicity of platelet activation mechanisms depending on the *Escherichia coli* strain testifies to the complexity of the interactions. Indeed, the mechanism involved seems to vary according to the virulence factors possessed by the strain. In the study by Watson et al., activation of FcγRIIA was in close collaboration with integrin-dependent αIIbβ3 signaling involved in platelet aggregation induced by some Gram-positive bacteria, notably Staphylococci (Cox et al., 2011).

Other phenomena have also been highlighted to explain the platelet activation occurring during sepsis, which would in fact be multifactorial and not solely dependent on direct activation by bacteria. The appearance of endothelial cell lesions during sepsis would favor not only inflammatory but also thrombotic phenomena (Opal and van der Poll, 2015; Piotti et al., 2021). These endothelial lesions can lead to the appearance of platelet activation signals, which contribute to the phenomenon of aggregation *in vivo*. Indeed, the endothelium will interact with certain bacterial structures, leading to its activation, the release of numerous molecules (pro-inflammation cytokines, chemokines, pro-coagulant factors) as well as the expression of adhesion molecules (VCAM-1, PECAM), favoring interactions with the figurative elements of the blood, including platelets (Joffre et al., 2020). The increase in these interactions, associated with an imbalance in the anticoagulant and antiaggregant function of the endothelium, would therefore increase the risk of thrombosis (Ince et al., 2016).

The anti-infective activity of platelets gives them a bactericidal power, which can be based on two mechanisms: indirect interaction with immune cells, or direct secretion by platelets of peptides with antimicrobial activity. These molecules belong to the platelet microbicidal peptide (PMP) family (Yeaman, 2014; Li et al., 2020), which includes CXCL4, CXCL7 (also known as PBP), and CCL5, but also the class of defensins [human β-defensin 2 (BD2)]; thymosin β4 (Tβ4) and derivative antimicrobial peptides (thrombocydins, fibrinopeptide A; Krijgsveld et al., 2000; Pasupuleti et al., 2012; Aquino-Domínguez et al., 2021). In a recent study, we demonstrated that the bactericidal activity of platelets was heterogeneous and depended on the *Escherichia coli* strain involved: out of ten strains tested, only three induced bactericidal activity from platelets. Comparison of the genomes of two strains with different behaviors revealed the existence of differences in the cluster of genes involved in O antigen synthesis.

Based on our results and the literature, we hypothesized that the structural variations of LPS could alter the interactions with platelets and lead to a loss of the ability of platelets to activate and induce a bactericidal response. The loss of this bactericidal mechanism induced by platelets secondary to a modification of LPS would thus be similar to what can be observed during resistance to certain antibiotics and would rely on the same mechanisms (Baron et al., 2016). However, few studies have looked at whether or not there is a link between antibiotic resistance and the ability of strains to interact with platelets. In our recent publication, we have shown that there appeared to be no link between colistin resistance and the ability of platelets to induce bacterial growth reduction using ten *Escherichia coli* strains (Ezzeroug Ezzraimi et al., 2022b).

There will, therefore, be a balance between the beneficial and deleterious effects of platelets during sepsis (Assinger et al., 2019). They will have a direct antimicrobial effect, tissue repair capabilities and will allow immunomodulation of the immune response as well as some chemotaxis. However, uncontrolled and disseminated activation can lead to the aggravation of sepsis, through the thrombotic and hemorrhagic risk that DIC can induce. The use of molecules capable of reducing this state of platelet hyperactivation could therefore have a beneficial effect on mortality during sepsis.

## Effect of antiplatelets in sepsis

Platelet count is included in the SOFA score and is inversely associated with the severity of sepsis (Hui et al., 2011). The severity of thrombocytopenia is often used to stratify patients with sepsis and septic shock. In general, 20–58% of septic patients develop thrombocytopenia, of which 10% develop severe thrombocytopenia (Thiolliere et al., 2013).

Many mechanisms have been proposed to explain thrombocytopenia in sepsis. A combination of several mechanisms remains the most likely hypothesis. Among other things, immune-mediated platelet activation decreases platelet life span, as activated platelets are rapidly cleared from the circulation (Aslam et al., 2006). Thus, reducing platelet activation could be a therapeutic target of interest for the prevention of morbidity and mortality in affected patients (Assinger et al., 2019). One fairly obvious hypothesis would, therefore, seem to be that, by reducing platelet reactivity, it would be possible to reduce their interactions with pathogens and the resulting consequences. Antiplatelets could, therefore, theoretically play an interesting role in improving the clinical prognosis during sepsis.

**Table 1:** Summary of data available in the literature concerning interactions between bacteria and platelets.

	Strains	Platelet receptor involved	References
*Escherichia coli*	O111	TLR4	Petruzziello-Pellegrini et al. (2013)
	O157:H7	FcγRIIA	Moriarty et al. (2016)
	O157	TLR4 CD62	Ståhl et al. (2009)
		
	CFT073 (O6:H1)	FcγRIIA αIIbβ3 integrin	Watson et al. (2016)
	RS218 (O18:H7:K1)	FcγRIIA αIIbβ3 integrin	Watson et al. (2016)
		
	K12 C600	αIIbβ3 integrin TLR4	Clark et al. (2007), Zhang et al. (2009), Lopes Pires et al. (2017), Fejes et al. (2018), Vallance et al. (2019)
	O111:B4	TLR4	Vallance et al. (2019)
	O55	TLR4	Zhang et al. (2009)
*Staphylococcus aureus*		αIIbβ3 integrin GPIbα	Cox (2009), Cox et al. (2011)
*Staphylococcus lugdunensis*		αIIbβ3 integrin	Cox (2009), Cox et al. (2011)
*Streptococcus pneumoniae*		TLR2	Cox (2009), Cox et al. (2011)
*Helicobacter pylori*		vWF	Cox (2009), Cox et al. (2011)

Several antiplatelet agents (APAs) are available that have a specific action on one of the platelet activation pathways. The most commonly used APAs are aspirin, which inhibits the synthesis of thromboxane A_2_ (TxA_2_), and inhibitors of the P_2_Y_12_ pathway (clopidogrel, prasugrel, and ticagrelor). Other antiplatelet agents, notably the anti-GpIIbIIIa, specifically inhibit platelet aggregation, have more limited indications and have been little evaluated, to our knowledge, in this indication.

Although many studies have evaluated the potential benefit of APAs in sepsis, the data of interest were presented in two meta-analyses and one literature review that aimed to determine whether APA administration had a beneficial effect on reducing the risk of mortality in sepsis (Zhao et al., 2002; Berthet et al., 2010; Vallance et al., 2017). The first meta-analysis included 15 studies conducted between 2011 and 2016, and concluded that there was a reduction in the risk of mortality of an average of 7%, ranging from 2 to 12% when aspirin was taken before the development of sepsis (Trauer et al., 2017). The analysis by Ouyang et al., published in 2019, includes 10 studies, of which four were also analyzed by Ouyang et al. (2019).

In both meta-analyses, the authors point to significant heterogeneity between studies. Indeed, although the results presented for each study were those of the subset of patients with sepsis, some of the studies included in these meta-analyses not only looked at cohorts of patients with sepsis, but also examined the effect of aspirin on the development of acute respiratory distress syndrome (ARDS; Erlich et al., 2011; Kor et al., 2016) or acute community-acquired pneumonia (Lösche et al., 2012; Falcone et al., 2015). Similarly, some studies looked at the effect of long-term aspirin use on the development of the acute episode, while others assessed its effect on mortality during hospitalization. However, the benefit of the administration of APAs and aspirin in particular is retained by these two meta-analyses.

A review of the literature published in 2018 by Wang et al. (2018) includes eight retrospective studies conducted between 2012 and 2016. Some of these conclude that there is a reduction in mortality when aspirin is taken in the ICU (Eisen et al., 2012; Lösche et al., 2012), while others do not show statistically significant benefits (Campbell et al., 2015). Conversely, only one study showed an increased risk of developing severe sepsis in the ICU for patients who are given aspirin (Al Harbi et al., 2016).

The two meta-analyses (Trauer et al., 2017; Ouyang et al., 2019) would therefore be in favor of a beneficial effect of aspirin in terms of mortality, whereas the review of the literature by Wang et al. is more reserved as to the conclusions of the effect of aspirin (Wang et al., 2018).

It is important to note that these clinical studies on the effect of APAs do not assess their potential benefit in relation to the bacterial species responsible for the septic condition. Some studies that have looked specifically at the Staphylococcus family have shown that aspirin and ticagrelor have a greater effect on *Staphylococcus sanguinis* than on *Staphylococcus aureus* (Hannachi et al., 2020). The effect of antiplatelet agents therefore appears to be variable depending on the bacterial species.

The retrospective study by Osthoff et al. (2016) is unique in that it only looked at the effect of aspirin in sepsis caused by *Staphylococcus aureus* in hospitalized patients with or without long-term low-dose aspirin therapy. Interestingly, the control population consisted of patients with *Escherichia coli* sepsis. The results indicated a significant reduction in mortality with aspirin in the *Staphylococcus aureus* sepsis group. In the *Escherichia coli* sepsis group, no reduction in mortality was observed with aspirin use. However, they did not necessarily attribute this result to treatment failure but to a much lower mortality rate than in the *Staphylococcus aureus* group, with a lack of statistical power to assess this parameter (Osthoff et al., 2016). In view of these data, it is difficult to confirm a possible beneficial role for aspirin in *Escherichia coli* sepsis.

However, a 2017 randomised study looked at the effect of antiplatelets when healthy subjects were given purified LPS from *Escherichia coli* O:113 (Kiers et al., 2017). Taking aspirin for 7 days before the administration of LPS would lead to an increase in the inflammatory response, by increasing the production of pro-inflammatory cytokines without, however, affecting the production of anti-inflammatory cytokines. However, dual therapy, with the addition of a P_2_Y_12_ inhibitor, reduces TNF-α production to levels comparable to those observed with placebo without, however, reducing the production of other pro-inflammatory cytokines. Platelets could therefore play a role in modulating the inflammatory response, in addition to their direct bactericidal effect, which could be modified by the administration of antiplatelets, and in particular aspirin. One of the reasons for the variability of the platelet response upon contact with different bacterial strains may be the affinity of the binding to TLR4 and the formation of the TLR4-sCD14 complex. Antiplatelets will also play an important role in this signaling pathway. sCD40L, released from platelet granules, is able to induce an increase in the secretion of thromboxane A_2_, and to potentiate the capacity of platelets to aggregate. Aspirin intake would inhibit this thromboxane A_2_ secretion without affecting CD40L levels, thereby fully or partially suppressing the potentiating effect of platelet aggregation in response to low doses of thrombin or collagen (Kojok et al., 2020). Aspirin is also thought to inhibit the phosphorylation of Myosin Light Chain (MLC), a protein involved in modifying the actin cytoskeleton structure of platelets, allowing them to remain in a resting conformation. CD40L levels in patients treated with different antiplatelet agents, shows that CD40L levels are indeed not significantly altered when taking aspirin, but that these levels are lowered when taking a P_2_Y_12_ inhibitor (Judge et al., 2005). A decrease in the membrane expression levels of P-selectin and GpIIbIIIa was also observed (Gremmel et al., 2015).

In sepsis, CD40L has been used as a possible marker of platelet activation being statistically higher in a group of patients admitted to intensive care compared to a control group (Vardon-Bounes et al., 2021). These results confirm that sCD40L could be an important prognostic marker in sepsis, with not only increased levels in septic patients, but also a significant association with mortality (Lorente et al., 2011).

Although the vast majority of studies on the effect of antiplatelets in sepsis have focused on the effect of aspirin and P2Y12 ADP receptor inhibitors, other molecules with a different mechanism of action could play an interesting role. This is particularly the case with inhibitors of the PAR-1 (Protease-activated Receptors) to thrombin, such as vorapaxar and atopaxar, which are still in development (de Souza and Tricoci, 2013), and which would therefore make it possible to obtain a reduction in thrombotic events secondary to thrombin-related platelet activation, without any consequence on normal hemostasis. However, although there are few data on the effect of these two molecules during sepsis, it was shown in a randomised double-blind trial that vorapaxar was able to induce a decrease in certain coagulation markers, notably the concentration of prothrombin F1 + 2 fragments and thrombin–antithrombin complexes (TAT) after injection of LPS into healthy subjects (Schoergenhofer et al., 2018). This molecule was also capable of inducing a decrease in the levels of antigenic Willebrand factor (vWF: Ag) and certain pro-inflammatory cytokines, notably TNF- and IL-6. Vorapaxar would therefore have a beneficial effect during sepsis, not only by reducing platelet activation, but also by its ability to act on other cell types. Monocytes play a fundamental role in the activation of coagulation during sepsis, notably through the significant release of tissue factor (TF), which is thought to be secondary to the activation of PAR-1 receptors on their surface (Pernerstorfer et al., 1999; Derhaschnig et al., 2004). If we look more specifically at the effect of these PAR-1 inhibitors during an *Escherichia coli* infection, we find a study that was particularly interested in the effect of one of these molecules, SCH79797, which not only induces an intense formation of NETs, but also has a direct antibiotic effect against the outer membrane of *Escherichia coli* (Aslam et al., 2006). However, these effects would not be found with vorapaxar, and would therefore not be dependent on PAR-1 inhibition.

## Conclusion

The interaction between platelets and bacteria is a complex mechanism, varying according to the species, and even according to the different strains belonging to the same species. This is the case for *Escherichia coli*. Some strains show low levels of interaction with platelets and induce platelet aggregation with a low probability. Conversely, other strains, recognized with greater affinity by certain platelet receptors, may expose the patient to an increased thrombotic risk, due to their ability to induce platelet aggregation.

Antiplatelets may play an important role in the management of sepsis, particularly in *Escherichia coli*. However, although the use of this class of drugs in this indication has been widely published, almost none of these studies have evaluated the benefit of antiplatelet drugs in sepsis according to bacterial species.

In order to assess the pro-aggregation potential of *Escherichia coli* strains, the determination of specific markers present on *Escherichia coli* would allow the prediction of the capacity of this strain to interact with platelets. Thus, with regard to the potential role of the O antigen in these reactions, its *in silico* serotyping would allow the rapid determination of the serotype of the strain involved from the FASTA sequencing data (Joensen et al., 2015; Bessonov et al., 2021).

The ability or inability to induce platelet aggregation for each of these strains could also be evaluated *in vitro*, in order to identify the strains and serotypes with a strong capacity to activate platelets. The identification of these strains would allow a more precise identification of the signaling pathways involved in platelet activation but would also make it possible to test each of the antiplatelet agents in order to assess which molecule is able to act in an optimal way to reduce platelet reactivity during sepsis.

Clinical studies are also needed to confirm the various results obtained *in vitro*, and to evaluate the clinical efficacy of antiplatelets in real-life conditions.

## Author contributions

All authors listed have made a substantial, direct, and intellectual contribution to the work and approved it for publication.

## Conflict of interest

The authors declare that the research was conducted in the absence of any commercial or financial relationships that could be construed as a potential conflict of interest.

## Publisher’s note

All claims expressed in this article are solely those of the authors and do not necessarily represent those of their affiliated organizations, or those of the publisher, the editors and the reviewers. Any product that may be evaluated in this article, or claim that may be made by its manufacturer, is not guaranteed or endorsed by the publisher.

## Abbreviations

APA: antiplatelet agent
ADP: adenosine diphosphate
CCL5: chemokine ligand 5
CXCL4: platelet factor 4
DIC: disseminated intravascular coagulation
ClfA: clumping factor A
C4BP: C4 binding protein
EHEC: Enterohaemorrhagic *Escherichia coli*
EIEC: Enteroinvasive *Escherichia coli*
EPEC: Enteropathogenic *Escherichia coli*
ETEC: Enterotoxigenic *Escherichia coli*
ExPEC: Extraintestinal pathogenic *Escherichia coli*
FcγRIIA: immunoglobulin Fc fragment receptor IIa
TF: tissue factor
IgG: immunoglobulin G
LPS: lipopolysaccharide
MODS: Multiple organ dysfunction syndrome
NET: neutrophil extracellular traps
NO: nitrogen monoxide
PAF: platelet activating factor
PAF-R: PAF receptor
PI3K: phosphoinositide 3-kinase
PSGL-1: P-selectin glycoprotein ligand-1
RR: relative risk
sCD14: soluble CD14
ARDS: acute respiratory distress syndrome
HUS: hemolytic uremic syndrome
SIRS: systemic inflammatory response syndrome
TLR4: Toll-like receptor 4
TxA2: Thromboxane A_2_
vWF: von Willebrand factor
